# Supplementary material for: Role of 5-HT2A, 5-HT2C, 5-HT1A and TAAR1 Receptors in the Head Twitch Response Induced by 5-Hydroxytryptophan and Psilocybin: Translational Implications
Source: Int J Mol Sci. 2022 Nov 16;23(22):14148. doi: 10.3390/ijms232214148 (PMC9698447; doi:10.3390/ijms232214148)
Supplement: Supplementary file 1 [file ijms-23-14148-s001.zip › Supplementary Data.pdf]

Shahar et al

Detailed Statistical Data

Supplementary Table S1. Detailed statistical results from figures.

Figure	Statistical Test	Details	Statistical Result
Figure 1A	Two-way ANOVA		F [5, 23] = 8.328, p = 0.0001
		time	F [3.830, 77.09] = 9.051, p < 0.0001
		time x dose	F [70, 322] = 2.031, p < 0.0001
Figure 1B	One-way ANOVA		F [5,23] = 8.328, p = 0.0001
	Post hoc: Dunnett	Vehicle vs. 5-HTP 150 mg/kg	p = 0.0343
	Post hoc: Dunnett	Vehicle vs. 5-HTP 200 mg/kg	p = 0.0002
	Post hoc: Dunnett	Vehicle vs. 5-HTP 250 mg/kg	p = 0.0204
Figure 1C	Nonlinear regression	EC50	101.6
		HillSlope	4.75
		R squared	0.5676
Figure 2A	One-way ANOVA		F [2,14] = 15.65, p = 0.0002
	Post hoc: Dunnett	Vehicle vs. M100907 0.5 mg/kg	p = 0.0007
	Post hoc: Dunnett	Vehicle vs. M100907 2 mg/kg	p = 0.0004
Figure 2B	One-way ANOVA		F [2,36] = 8.385, p = 0.0010
	Post hoc: Dunnett	Vehicle vs. 8-OH-DPAT 1 mg/kg	p = 0.0035
	Post hoc: Dunnett	Vehicle vs. 8-OH-DPAT 2 mg/kg	p = 0.0014
Figure 2C	One-way ANOVA		F [3,13] = 7.229, p = 0.0042
	Post hoc: Tukey	Vehicle vs. RS102221 4 mg/kg	p = 0.0059
	Post hoc: Tukey	Vehicle vs. RS102221 8 mg/kg	p = 0.0176

Figure 2D	One-way ANOVA		F [2,11] = 4.939, p = 0.0295
	Post hoc: Dunnett	Vehicle vs. EPPTB 1 mg/kg	p = 0.0421
	Post hoc: Dunnett	Vehicle vs. EPPTB 10 mg/kg	p = 0.0344
Figure 3A	Two-way ANOVA		F [15, 83] = 7.132, p < 0.0001
		Time	F [3.282, 272.4] = 37.12, p < 0.0001
		Time x dose	F [135, 747] = 9.036, p < 0.0001
Figure 3B	Two-way ANOVA	Time	F [2.882, 250.8] = 56.66, p < 0.0001
		Dose group	F [1, 87] = 20.79, p < 0.0001
		Time x Dose group	F [9, 783] = 81.78, p < 0.0001
Figure 3C	One-way ANOVA		F [16,88] = 7.156, p < 0.0001
	Post hoc: Dunnett	Vehicle vs. PSIL 0.5 mg/kg	p = 0.0085
	Post hoc: Dunnett	Vehicle vs. PSIL 0.75 mg/kg	p = 0.0194
	Post hoc: Dunnett	Vehicle vs. PSIL 0.8 mg/kg	p = 0.0296
	Post hoc: Dunnett	Vehicle vs. PSIL 1.6 mg/kg	p < 0.0001
	Post hoc: Dunnett	Vehicle vs. PSIL 3 mg/kg	p = 0.0007
	Post hoc: Dunnett	Vehicle vs PSIL 3.2/4.4/6.4 mg/kg	p < 0.0001
	Post hoc: Dunnett	Vehicle vs PSIL 12.8 mg/kg	p = 0.008
	Post hoc: Dunnett	Vehicle vs PSIL 25.6 mg/kg	p < 0.0001
Figure 3D	One-way ANOVA		F [15,107] = 20.05, p < 0.0001
	Post hoc: Dunnett	Vehicle vs. PSIL 1.6 mg/kg	p = 0.0138
	Post hoc: Dunnett	Vehicle vs. PSIL 3 mg/kg	p= 0.0007
	Post hoc: Dunnett	Vehicle vs. PSIL 3.2/4.4/6.5/12.8/25.6 mg/kg	p < 0.0001
Figure 3E	Nonlinear regression	EC50	2.588
		HillSlope	1.026
		R squared	0.6894
Figure 4A	One-way ANOVA		F [4,34] = 35.03, p < 0.0001

	Post hoc: Dunnett	Vehicle vs. M100907 0.5/2 mg/kg	$p < 0.0001$
	Post hoc: Dunnett	Vehicle vs. 8-OH-DPAT 1/2 mg/kg	$p < 0.0001$
Figure 4B	One-way ANOVA		$F [3,24] = 8.178$, $p = 0.0006$
	Post hoc: Tukey	Vehicle vs. RS102221 4 mg/kg	$p = 0.0363$
	Post hoc: Tukey	RS102221 2 mg/kg vs. RS102221 4 mg/kg	$p = 0.0371$
	Post hoc: Tukey	RS102221 4 mg/kg vs. RS102221 8 mg/kg	$p = 0.0006$
Figure 5	Two-way ANOVA	Compound	$F [1, 8] = 6.099$, $p = 0.0387$
		Time x Compound	$F [14, 112] = 13.91$, $p < 0.0001$

Shahar et al

Magnetometer Apparatus

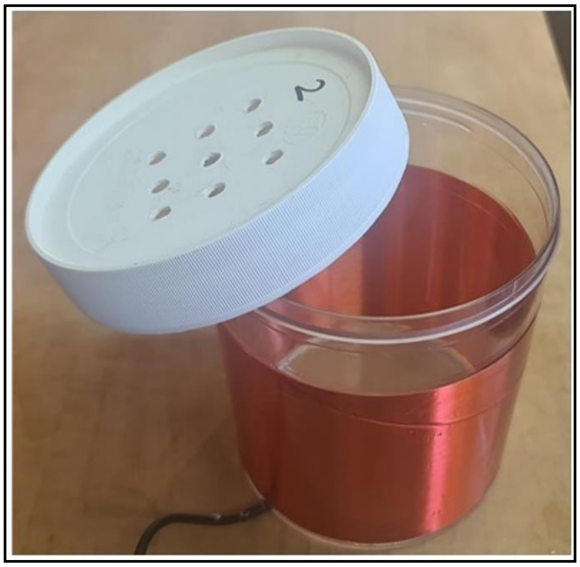

Supplementary Figure S2. Magnetometer apparatus that was used to measure HTR. One of the six magnetometer coils that make up the system.

Shahar et al

Magnetometer Validation

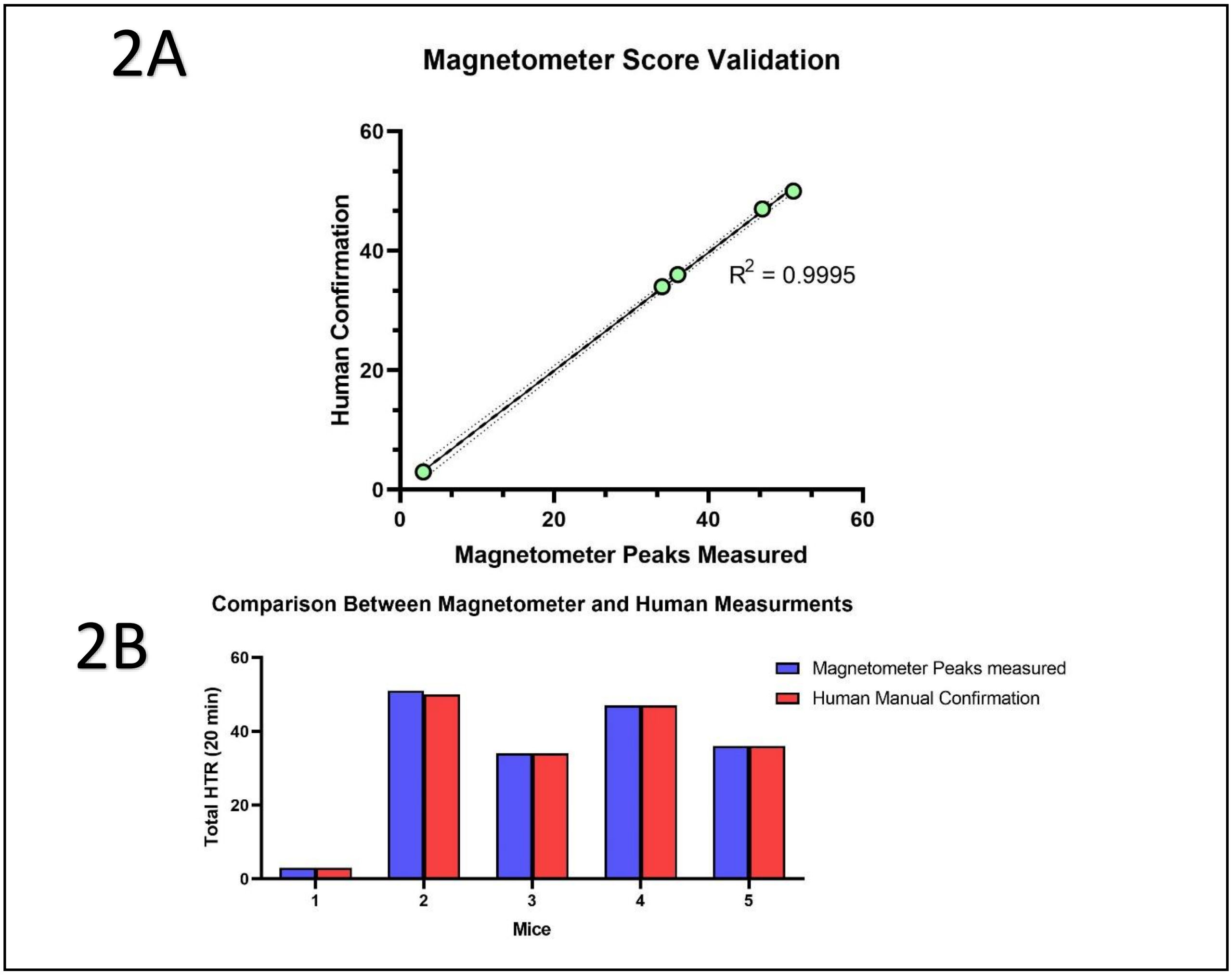

Supplementary Figure S2.

(A) Validation magnetometer peak scores showed high correlation by human scoring of video of the same mouse while in the magnetometer apparatus (mice n=5, total peaks counted n=307). Simple linear regression: Y = 0.9882*X + 0.2038, R^2 = 0.9996. F [1,3] = 6928, P < 0.0001. (B) Bar graph representation of the simple linear regression (A).
